# Effect of Elevated Ambient Temperature on Maternal, Foetal, and Neonatal Outcomes: A Scoping Review

**DOI:** 10.3390/ijerph19031771

**Published:** 2022-02-04

**Authors:** Yohani Dalugoda, Jyothi Kuppa, Hai Phung, Shannon Rutherford, Dung Phung

**Affiliations:** 1 School of Medicine and Dentistry, Griffith University, Brisbane, QLD 4222, Australia; jyothirmaye.kuppa@gmail.com (J.K.); hai.n.phung@griffith.edu.au (H.P.); s.rutherford@griffith.edu.au (S.R.); 2 School of Public Health, The University of Queensland, Brisbane, QLD 4006, Australia; d.phung@uq.edu.au

**Keywords:** elevated ambient temperature, maternal foetal and neonatal outcomes, exposure windows, temperature metrices, environmental risk factor, scoping review

## Abstract

This scoping review provides an overview of the published literature, identifies research gaps, and summarises the current evidence of the association between elevated ambient temperature exposure during pregnancy and adverse maternal, foetal, and neonatal outcomes. Following the PRISMA extension for scoping reviews reporting guidelines, a systematic search was conducted on CINAHL, PubMed, and Embase and included original articles published in the English language from 2015 to 2020 with no geographical limitations. A total of seventy-five studies were included, conducted across twenty-four countries, with a majority in the USA (*n* = 23) and China (*n* = 13). Study designs, temperature metrics, and exposure windows varied considerably across studies. Of the eighteen heat-associated adverse maternal, foetal, and neonatal outcomes identified, pre-term birth was the most common outcome (*n* = 30), followed by low birth weight (*n* = 11), stillbirth (*n* = 9), and gestational diabetes mellitus (*n* = 8). Overall, papers reported an increased risk with elevated temperature exposures. Less attention has been paid to relationships between heat and the diverse range of other adverse outcomes such as congenital anomalies and neonatal mortality. Further research on these less-reported outcomes is needed to improve understanding and the effect size of these relationships with elevated temperatures, which we know will be exacerbated by climate change.

## 1. Introduction

Climate change is the most significant global threat we face in the 21st century, and its health impacts are growing [1,2]. A changing climate is a key factor in increasing the intensity, duration, and frequency of extremes of heat and the associated exacerbation of existing health challenges for all populations, especially vulnerable population groups, such as the elderly, people with chronic disabilities, young children, newborn babies, and pregnant women [3,4,5,6,7].

There is evidence of heat stress as a risk for pregnant women due to (i) increased body weight, and body-fat-triggered increased core body temperature and heat production; (ii) decreased ratio of surface area to body mass associated with pregnancy, reducing the heat-loss capacity of sweating, and (iii) foetal metabolic rate increasing the maternal core body temperature [8,9]. When environmental temperature exceeds the maternal core body temperature, it causes cutaneous vasodilation and sweat secretion, and decreases uterine and umbilical cord blood flow [10]. If heat-loss mechanisms are inadequate to dissipate heat effectively, the body becomes dehydrated [11,12]. In such conditions, the endocrine system activates and releases the antidiuretic hormone and oxytocin, further decreasing uterine blood flow to the foetus and triggering labour, often prematurely [12]. Heat exposure can cause acute heat stress and release heat-shock protein [13]. Heat-shock protein damages placental cells and reduces placental efficiency, decreasing adequate oxygen and nutrition supply to the developing foetus, leading to low birth weight [13]. In addition, heat stress can interrupt the typical sequence of gene activity during organogenesis, leading to congenital anomalies, particularly during early stages of gestation or stillbirths at the later stages [14,15]. In addition, extreme heat exposure during pregnancy affects glucose metabolism and increases insulin resistance, leading to gestational diabetes [16,17,18]. Moreover, some evidence indicates that elevated temperature causes maternal hypertension, especially pre-eclampsia, one of the main risk factors for global maternal mortality [19].

These pregnancy-related physiological and anatomical changes associated with altered thermoregulation mechanisms not only make pregnant women vulnerable to increased temperatures at all stages of pregnancy but also are linked to adverse maternal, foetal, and neonatal outcomes including but not limited to preterm birth (PTB), low birth weight (LBW), stillbirth, congenital anomalies, gestation diabetes mellitus (GDM), hypertension disorders, and maternal stress [6,8,15,16,20,21,22,23,24].In particular, heat-associated adverse neonatal outcomes such as PTB, LBW, and stillbirth have been widely reported in the literature [9,20,21].

PTB, defined as a baby being born before 37 weeks of gestation, is a global epidemic with approximately 15 million global incidences every year [22,23,24]. PTB is a leading cause of childhood mortality and morbidity under five years and the direct cause of neonatal mortality (death within 28 days of births) [23]. In addition, LBW (live births under 2500g) is associated with prenatal mortality and morbidity and increases the risk of non-communicable diseases later in life [25]. Stillbirth is a birth following foetal death before labour or during labour, accounting for 2.0 million deaths globally in 2019 [26].

Despite research demonstrating the effect of elevated ambient temperature on specific adverse outcomes such as PTB, LBW, and stillbirth [9,21,27,28,29], the effect of elevated ambient temperature on other possible maternal, foetal, and neonatal adverse outcomes have often been neglected in current literature. Therefore, the magnitude and the trends of published literature on this topic to date are unclear. Furthermore, little attention has been paid to exploring susceptible windows and elevated temperature exposures. Therefore, this scoping review aims to describe the characteristics of the published literature, identify research gaps, and summarise the current evidence of the association between elevated ambienttemperature exposure during pregnancy and a range of adverse maternal, foetal, and neonatal outcomes. Specific research questions addressed in this scoping review include:

(1) What are the magnitude, characteristics, and trends of research on elevated ambient temperature and maternal, foetal, and neonatal outcomes?
(2) What adverse maternal, foetal, and neonatal outcomes are being explored by research for their relationships with elevated ambient temperature; what are the findings?
(3) What gestational periods are particularly susceptible to elevated ambient temperatures during pregnancy?

In addition, the review will identify the common limitations and gaps of published literature to inform recommendations for future studies.

This scoping review examined the elevated ambient temperature exposure (e.g., exposure to various high ambient temperatures, heatwave, and extreme temperature events) and adverse maternal, foetal, and neonatal outcomes regardless of the typical weather patterns to which pregnant women were exposed during their pregnancy. However, the review did not examine how the pregnant body would acclimate to higher temperatures.

## 2. Materials and Methods

### 2.1. Search Strategies

This study followed the PRISMA extension (Preferred Reporting Items for Systematic Reviews and Meta-Analyses) for scoping review reporting guidelines. The initial systematic search was conducted on CINAHL, PubMed, and Embase from 2005 to 2020. The keywords were developed referring to keywords from published original articles and previous systematic reviews with the assistance of a professional librarian at Griffith University.

A broad range of relevant key words were included based on (i) heat-related extreme events (search terms included: “heat wave” OR “extreme heat” OR “extreme temperature” OR “high temperature” OR “excessive temperature” OR “hot temperature” OR “heat exposure” OR “ambient temperature” OR “environmental temperature” OR “extreme weather” OR “hot weather” OR “warm weather” AND (ii) adverse maternal, foetal, and neonatal outcomes associated with heat (search terms included: “pregnan*” OR “foet*” OR “fetal” OR “fetus” OR “expectant mothers” OR “maternal health” OR “maternal morbidity” OR “gestational age*” OR “antenatal” OR “prenatal” OR “birth outcome” OR “pregnancy outcome” OR “stillbirth” OR “preterm” OR “premature” OR “prematurity” OR “birth weight” OR “eclampsia” OR “miscarriage” OR “abortion” OR “pre-eclampsia” OR “gestational hypertension” OR “placental abruption” OR “foetal death” OR “fetal death” OR “neonat*” OR “newborn”.

### 2.2. Inclusion/Exclusion Criteria and Identification of Relevant Studies

#### 2.2.1. Inclusion Criteria

- Studies that included pregnant women and/or neonates in the study population;
- Studies that directly addressed the relationship between elevated ambient temperatures (e.g., heatwave, extreme temperature, various high ambient temperatures), and pregnancy and birth outcomes in any stage of pregnancy;
- Studies that examined one or more maternal, foetal, or neonatal outcomes;
- Original research studies published in the English language;
- All types of study designs;
- Articles published between January 2005–November 2020 (the initial collection of articles generated using this time frame).

#### 2.2.2. Exclusion Criteria

- Studies that did not include pregnant women or neonates in the study population;
- Studies that showed associations between other indicators of climate change or heat sources such as hot baths, saunas, or experimental temperatures;
- Studies that did not examine adverse maternal, foetal, or neonatal health outcomes;
- Reviews and grey literature;
- Articles published in languages other than English language;
- Articles published before 2005.

All identified articles from the searches were transferred into EndNote, duplicates removed, and uploaded to Rayyan, a web tool designed to help expedite the article screening and selecting process [30]. Two reviewers (YD and JK) independently determined study eligibility in two phases: (i) title and abstract screening and (ii) full-text review using the inclusion/exclusion criteria mentioned above, and a third reviewer (DP) resolved conflicts.

### 2.3. Data Extraction

**Figure 1:**
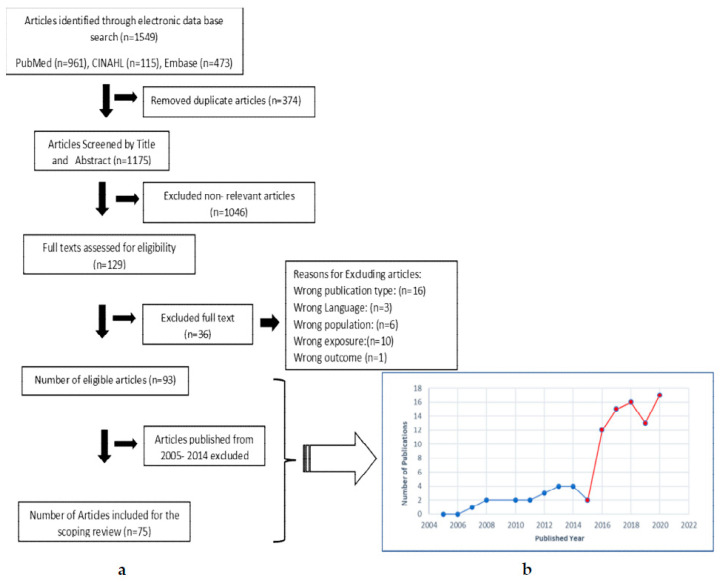
Article selection process. (**a**) Flow chart of article selection process. (**b**) Number of publications from 2005 to November 2020. The study initially searched articles published from January 2005 to November 2020 (**a**). However, after analyzing preliminary data of selected articles, the authors noticed a rapid increase in the number of publications after 2015 (**b**). Therefore, for this scoping review, the authors decided to restrict the analysis to articles published between 2015 to November 2020.

Eligible articles were extracted, tabulated, and checked for accuracy by the two reviewers (YD and JK). The following data were recorded: first author, publication year, duration of the study (start and end year), study title, study location, and sublocations, study objectives, study design type, study population (type, age range), number of participants, exposure measurement, outcome measurement, covariates examined, exposure window, study findings, a measure of effect, proposed mechanisms, limitations, and recommendations. Figure 1 describes the study selection process.

## 3. Results

### 3.1. The Magnitude, Characteristics, and Trends of Research on Elevated Ambient Temperature and Maternal, Foetal, and Neonatal Outcomes

The initial search of the three databases identified 1549 articles (PubMed: 961, Embase: 473, CINAHL: 115), of which 374 were duplicates, leaving 1175 articles for the title and abstract screening. Of these, 1046 were excluded, and 129 studies were selected for full-text review. From these, 36 articles were excluded due to their full text being unavailable, they were not in English, or they did not include any component of maternal exposure to elevated temperatures. A total of 93 studies were subjected to preliminary data analysis, and we observed a marked increase in the number of studies per year on this topic after 2015. As shown in Figure 1b, only eighteen studies were published before 2015 and 75 after that. Therefore, the 75 original articles published between 2015 to 2020 were selected for this scoping review because authors believe that this time frame provides the most up-to-date evidence.

**Figure 2:**
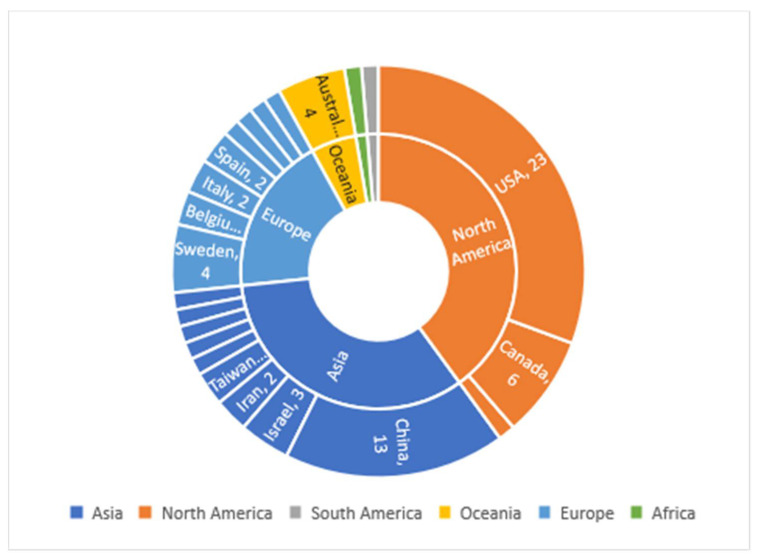
Geographic scope of all original articles reviewed.

The studies were performed across 24 countries globally, and most of them were conducted in North America (*n* = 30), Asia (*n* = 25), and Europe (*n* = 14) (Figure 2). The majority were single-country studies (*n* = 73), with two multi-country studies. As shown in Figure 2, a significant proportion of studies were conducted in the USA (*n* = 23) and China (*n* = 13).

Further analysis was conducted based on the income status of the countries. According to the World Bank’s current income status classification, countries were classified into low income, lower middle income, upper middle income, and high income [31]. The findings revealed that most single-country studies were conducted in high-income and upper- middle-income countries (95%), with only 5% in lower-middle-income countries and none in low-income countries.

All articles reviewed were quantitative; the retrospective cohort was the most used design to study the effect of heat exposure during pregnancy and adverse maternal, foetal, and neonatal outcomes (40%). Ecological and case-crossover studies were the second most common designs, accounting for nearly 16% each, while case-control and prospective cohort design was employed in 9% and 13% of included studies respectively. Finally, a cross-sectional design was the least common study design among reviewed studies.

Various temperature metrics were used to examine the influence of heat exposure on pregnant women and newborn babies (Table 1). Daily temperature measurements such as daily mean temperature (*n* = 35), daily maximum temperature (*n* = 26), and daily minimum temperature (*n* = 16) were the most common temperature metrics used among reviewed studies. Furthermore, studies calculated weekly, monthly, and trimester-specific exposure temperatures based on daily temperature measurements. Nine studies employed apparent temperature (combined effect of air temperature and relative humidity) to identify the exposure effects on elevated ambient temperatures on pregnant women and neonates [11,12,32,33,34,35,36,37,38]. Twelve studies examined the effects of heatwaves/extreme heat events on maternal, foetal, and neonatal outcomes [6,32,38,39,40,41,42,43,44,45,46,47]. They used different heatwave characteristics, i.e., length of exposure, threshold percentile (75th, 90th, 95th, and 98th percentile to identify high temperatures), peak temperature, and a number of heatwaves/heat events, to estimate the magnitude of the exposure effects [6,32,38,39,40,41,42,43,44,45,46,47]. No consistent definition for ‘heatwave’ or ‘extreme heat event’ was observed among reviewed studies.

Furthermore, more than 60% of studies reviewed focused on investigating the adverse maternal, foetal, or neonatal outcomes in warmer months or the summer season. Only 23% examined the exposure effects throughout the year (Table 2).

**Figure 3:**
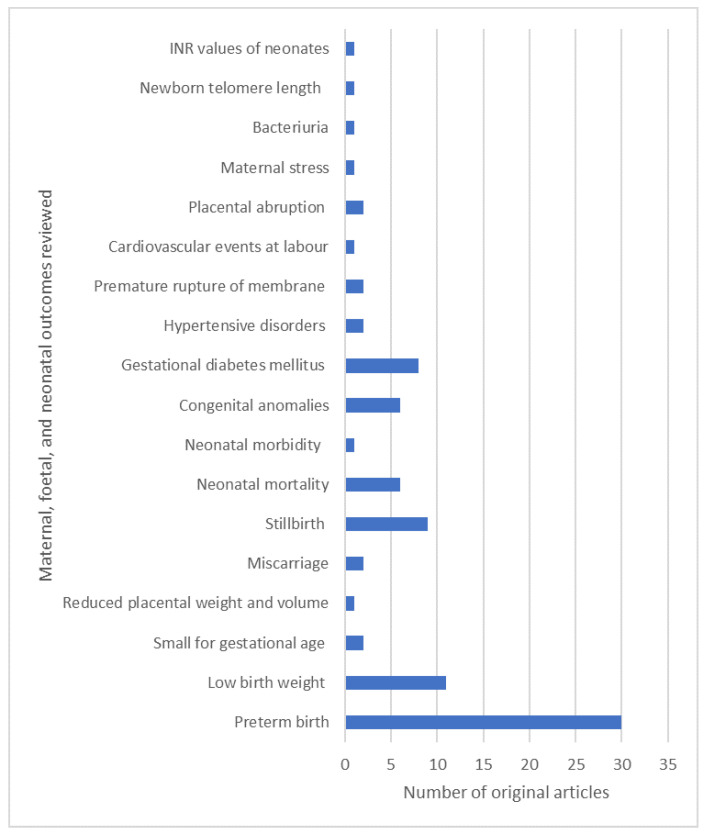
Maternal, foetal, and neonatal outcomes examined in this review. Note: Some studies examined multiple outcomes.

Reviewed studies covered 18 maternal, foetal, and neonatal outcomes related to elevated temperatures. PTB was the most common adverse outcome observed (*n* = 30) followed by LBW (*n* = 11), stillbirth (*n* = 9), and GDM (*n* = 8). Congenital anomalies, miscarriage, babies that are small for gestational age (SGA), neonatal morbidity, neonatal mortality, newborn telomere length, international normalisedratio (INR) values of neonates, maternal stress, bacteriuria, cardiovascular events, reduced placental weight and volume, placental abruption, premature rupture of membrane (PROM), and hypertensive disorders in pregnancy are the other reported adverse outcomes included in this review (Figure 3).

We observed that approximately 83% of included articles added a discussion of a proposed biological mechanism to explain their findings. Most of the included studies indicated that heat stress increases the risk of PTB, LBW, SGA, stillbirth, congenital anomalies, PROM, and GDM through various biological mechanisms [11,13,14,18,33,74]. For instance, heat-stress-associated hormonal changes induce labour and increase PTB occurrence [11,13,33,50,56,68,83]. Heat stress also releases heat-shock proteins, which damage placental cells, interrupts the typical sequence of gene activity during organogenesis, and inhibits cellular proliferation leading to stillbirths, miscarriages, and congenital anomalies [14,15,48,74]. In addition, heat-stress increases beta-cell dysfunction and insulin resistance; and reduces glucose uptake by insulin target tissues, leading to GDM [18]. In addition, several studies claimed a heat-associated maternal dehydration and impaired thermoregulation link to PTB, LBW, SGA, stillbirth, maternal hypertension disorders, placental abruption, and GDM [11,12,50,61,65,68,70,71,73,78,81,89]. In addition, a few studies argued that maternal exposure to prolonged sunlight promotes slower foetal growth [77], and that changes in sleeping patterns may be linked to maternal psychological stress [53].

### 3.2. Relationships between Elevated Ambient Temperature and Maternal, Foetal, and Neonatal Outcomes

#### 3.2.1. Elevated Ambient Temperature-Associated Adverse Neonatal Outcomes

##### Preterm Birth (PTB)

Among the 75 studies examined, PTB was the most common adverse outcome (*n* = 30) observed in this review. Among these, 23 studies reported that elevated temperatures significantly correlate with an increased risk or rate of preterm birth [11,12,13,32,33,39,43,44,46,50,52,56,58,59,61,63,65,69,77,79,80,92,93]. For instance, a time-series study conducted in Iran reported a significant increase in PTB risk from heatwave days (mean daily temperature > 90th percentile ≥ 2 consecutive days) compared to non-heatwave days (RR 1.21; 95%CI 1.08: 1.37) [32]. In addition, Smithand Hardeman [44] also reported a higher relative risk for PTB (RR 1.14; 95% Cl 1.0:1.3) in women exposed to heatwaves (≥37 °C) for a seven-day period of their pregnancy than those not exposed to a heatwave. Furthermore, a study conducted in Guangzhou, China, indicated that exposure to extreme heat (31.9 °C, the 99th percentile) increased the PTB risk by 10.0% [65]. Similarly, Ha et al. [63] reported that prenatal exposure to extreme heat during the first seven weeks of pregnancy increased the PTB risk by 11%, and that the entire pregnancy period increased PTB risk by 6%–21%. The study further indicated that exposure to a 5 °F (around 2.8 °C) increase in temperature during the last week of pregnancy increases the PTB risk by 12% to 16% in warm months [63]. However, a study conducted in Shenzhen, China, found a negative association between elevated temperature and premature birth, arguing that high ambient temperature might be a protective factor of PTB (RR 0.69 (at temperatures of 29.9 °C) and 0.62 (at temperatures of 30.7 °C)) [68]. One study found mixed effects of ambient temperature (>32 °C) on PTB, with temperature generally a protective factor or having no direct effect on PTB [90].

##### Low Birth Weight (LBW)

Eleven studies examined the relationship between maternal heat exposure and low birth weight at the time of delivery [10,13,35,41,55,71,77,78,88,92,94]. Of those studies, five found that elevated temperatures significantly reduce birth weight [10,35,41,77,78]. For instance, Poeran et al. [10] found that maternal exposure to summer temperatures (>37 °C) significantly reduced birth weight by 16–19 g. Likewise, Weng et al. [77] foundthat the incidence rate of LBW was highest in high temperatures (6.09% at 29.5 °C to around 30.8 °C) than in low temperatures (5.84% at 13.4 °C to around 15.4 °C). In addition, Lawrence et al. [41] reported that compared with pregnant women not exposed to extreme heat events(temperature ≥97th percentile of the maximum temperature (32.2 °C) for two consecutive days), those exposed were more likely to have a LBW baby. In addition, Molina and Saldarriaga [88] found that exposure to an increased temperature reduces birth weight by 20 g and increases the probability of a baby being born with low birth weight by 0.7%. Furthermore, Ngo and Horton [55] reported that exposure to an extra one day of average temperature greater than 85 °F (29.4 °C) in trimesters 1 and 2 reduces birth weight by 1.1 g (*p* < 0.10), where the cumulative impact during the entire pregnancy is associated with a reduction in birth weight of 1.7 g. However, Son et al. [92] found no statistically significant effect of ambient temperature on low birth weight.

##### Stillbirth

Nine studies examined the impact of maternal exposure to elevated temperature and stillbirth, and overall, papers reported an increased risk for stillbirth with elevated temperatures [15,36,37,45,49,52,77,91,95]. Weng et al. [77] found that the incidence of stillbirths is significantly associated with summer temperatures (>23.4 °C), and the incidence of stillbirths was highest at 29.4 °C. Li et al. [52] reported a significant increase in stillbirth risk related to the second trimester of pregnancy temperature levels (21.4 °C) (Hazard ratio 1.47, 95%CI: 1.24, 1.74). Two studies [15,91] reported that a 1 °C increase in ambient temperature during the last week of pregnancy was associated with a 6% and 7% increase in risk for stillbirth, respectively. Lastly, Basu et al. [36] and Rammah et al. [37] reported that a 10 °F (5.6 °C) increase in apparent temperature in the last week of pregnancy increased the stillbirth risk by 10.4% and 45%, respectively.

##### Neonatal Mortality

This review included six studies examining the association between elevated temperature and neonatal mortality [34,66,67,77,84,86].Two studies found an association between elevated temperatures and an increased risk of neonatal mortality [34].For instance, a US study of 9070 neonates found that increased apparent temperature was associated with a 4.6% increased risk for all-cause mortality and a 27% increased risk for respiratory mortality among neonates [34]. In addition, a Swedish study indicated that temperature increases from 14.5 °C to 20 °C were associated with a 25% increase in neonatal mortality [67]. Furthermore, this association increased steeply with increased temperatures, resulting in an almost 50% higher risk at temperature greater than 24 °C [67]. In contrast, two studies found elevated temperature led to lower mortality in neonates [66,84]. For instance, a study examining the risk of sudden infant death syndrome of neonates and post neonates indicates that heat-related sudden infant death syndrome risk is lower among neonates than postneonates [66]. Similarly, a study conducted in Bangladesh observed that hotter temperatures (>40 °C) led to lower mortality in neonates [84]. However, two studies reported that elevated temperature had no impact on neonatal mortality [77,86].

##### Neonatal Morbidity

The relationship between various neonatal morbidities and heatwaves was reported only in one study [6].The study found that neonates exposed to heatwaves while in utero are more likely to suffer from foetal distress, ventilator-assisted breathing for more than 30 min, and meconium aspiration syndrome [6]. However, no statistically significant results were reported for the morbidities mentioned [6].

##### Small for Gestational Age (SGA)

Neonates that are small for gestational age are those that have lower than expected weight (birth weightless than the 10th percentile of neonates with the same gestational age), length, and head circumference for gestational age [71,94]. SGA has been associated with an increased risk for stillbirth and neonatal mortality [71,94]. However, to date, there is limited research on this topic. This review identified only two studies that evaluated the high temperature and SGA relationship, and their findings were inconsistent. For instance, one study conducted using 29,597,735 term births across 403 US counties showed that exposure to temperatures warmer than average temperatures(above the 90th percentile) during the entire pregnancy was associated with an increased risk of term SGA (OR = 1.041) [71]. However, the other study, with 220,572 births across 12 US sites, found no association between high temperatures (above 95th percentile) and SGA during the trimester-specific windows of pregnancy (first and second trimesters) or during the entire pregnancy [94].

##### International Normalised Ratio (INR) of Neonates

One study investigated the seasonal variation and effects of elevated temperature on blood coagulation in neonates [51]. The study observed significantly higher INR values in summer (1.11 ± 0.10)than in winter (1.06 ± 0.09) in neonates [51]. Furthermore, the study found a significant correlation between outdoor high ambient temperature and the INR values suggesting that outdoor temperature was the most influential factor on the INR values of neonates [51].

##### Newborn Telomere Length

Martens et al. [82] reported that higher temperature (>19.5 °C) was associated with shorter cord blood telomere length. This parameter is essential for cellular function, aging, and disease susceptibility over the lifespan. The study found that the association was strongest with a 1 °C increase in temperature at week 36 of gestation, resulting in a 3.29% increase in shorter cord blood telomere length [82]. However, no significant association was observed for postnatal heat exposures [82].

#### 3.2.2. Elevated Ambient Temperature-Associated Adverse Foetal Outcomes

##### Congenital Anomalies

Out of 75 studies, 6 reported congenital anomalies and prenatal temperature exposure relationships, but the findings were inconsistent [14,38,42,47,48,85]. Three studies evaluated the association between high temperatures and congenital heart defects (CHD) [42,47,48]. For instance, a study conducted in Quebec, Canada, found that maternal exposure to a maximum daily temperature of ≥30 °C was significantly associated with an increased risk of multiple and noncritical congenital heart defects, particularly atrial septal defects (RR 1.37, 95%CI: 1.10,1.70) [48]. The study further reveals that the risk for noncritical CHD further increased with extreme summer heat exposures [48]. However, the other two studies found an increase in specific CHD subtypes during spring. For instance, Zhang et al. [47] reported a 34.0% increase in conotruncal CHD and a 38.6% increase in an atrial septal defect during spring. In addition, Lin et al. [42] reported extreme heat events in spring significantly associated with conotruncal defects and ventricular septal defects (ORs 1.23–1.78). Studies further highlighted that these associations increased steadily with more days exposed to elevated ambient temperature [42,47].The other three studies examined the maternal temperature exposure risk on neural tube defects, hypospadias, and orofacial clefts (OFCs) [14,38,85]. However, only neural tube defects show a weak positive association with high temperatures (>30 °C), suggesting that elevated temperature may be a risk factor for neural tube defects [14].

##### Reduced Placental Weight and Volume

The placenta plays an essential role in ensuring the normal growth of the foetus [96]. Reduced placental weight and volume are associated with adverse neonatal and maternal outcomes such as LBW and preeclampsia [96]. One study examined in this review reported a negative association between high temperatures and placental weight and volume and a positive association with placental efficiency [75].The study observed that maternal exposure to elevated temperature (29 °C) during late pregnancy reduces placental weight by 6.03 g, placental volume by 16.15 cm^3^, and increases placental efficiency by 0.26 [75].

##### Miscarriage

Only two included studies examined the effect of elevated temperature on miscarriage [74,95]. A study conducted in Guangdong, China, found that exposure to moderately high temperatures (23.1 °C) during the last two months before hospitalisation increased the risk of miscarriage (OR 1.243) before 28 weeks gestation [74]. The findings of the other study also suggested a possible association between elevated temperature and miscarriages, but the results were not statistically significant [95].

#### 3.2.3. Elevated Ambient Temperature-Associated Adverse Maternal Outcomes

##### Gestational Diabetes Mellitus (GDM)

Gestational diabetes mellitus is the most observed adverse maternal outcome among pregnant women among the reviewed studies. Eight studies evaluated the association between temperature variables and GDM [16,17,18,54,60,70,73,89].Despite variations in geographical location and seasonal definitions, all studies consistently reported an increased prevalence of GDM, the likelihood of GDM diagnosis, and serum glucose levels with elevated temperatures, especially in the summer season [16,17,18,54,60,70,73,89].

For instance, a Canadian cohort study that observed 555,911 births among 396,828 pregnant women over 12 years found a direct relationship between mean 30-day outdoor air temperature(>24 °C) before the routine GDM screening at 27 weeks of gestation and the likelihood of being diagnosed with GDM [60]. The study further revealed that each 10 °C increase in mean 30-day temperature was associated with a 6–9% relative increase in the risk of GDM [60]. Furthermore, a study conducted in Taiwan detected a rapid increase in GDM prevalence with temperatures greater than 28 °C [73].

In addition, a few studies reported higher serum glucose levels on the day of the oral glucose tolerance test (OGTT—the most used method of GDM screening) in summer [16,17]. For instance, a study conducted in Italy (*n* = 5473) observed that blood glucose levels at one hour and two hours after administrating 75 g OGTT were significantly higher in summer than in spring, autumn, and winter [16]. Similarly, a Sweden cohort (*n* = 11,538) observed a mean two-hour blood glucose level increase by 0.009 mmol/L for a 1 °C rise in temperature [17]. Vasileiou et al. [89] reported significantly higher three-hour OGTT glucose levels in summer among pregnant women in Greece. Molina-Vega et al. reported the association between the higher mean ambient temperature on the day of OGTT and 14–28 days before the OGTT increased the risk of GDM among pregnant women in Spain [54]. The remaining studies reported increased odds or risk of GDM with various elevated ambient temperature variables [18,70].

##### Hypertensive Disorders

Two studies examined the association between elevated temperature and hypertensive disorders during pregnancy [6,19].Both studies consistently reported that maternal exposure to heatwaves [6] and high average temperature [19] increased the risk of preeclampsia, eclampsia, and gestational hypertension.

##### Premature Rupture of Membrane (PROM)

PROM occurs due to the natural weakening of the foetal membrane, which triggers the foetal membrane’s rupture without labour onset [64,72]. Two studies examined the association between ambient temperatures and PROM [64,72]. Both showed that elevated temperatures are associated with a higher risk of PROM [64,72]. For instance, a US study (*n* = 15,381 singleton pregnancies) reported that each 1 °C increase in ambient temperature during the seven days before the delivery was associated with a 5% and 4% increase in preterm PROM risk and term PROM risk, respectively [64]. Similarly, the Chinese study that observed 3255 PROM cases among 29,608 single births found that exposure to extreme temperature (32 °C, 99th percentile) was significantly associated with a 2.161 times relative risk of PROM at a lag of 0–2 days [72].

##### Placental Abruption

Two studies reported a high risk for placental abruption associated with high-temperature exposures during the pregnancy period [37,81]. For instance, research conducted in Canada reported that elevated temperature (>30 °C) in warm seasons increases the risk of placental abruption by 7%, particularly for women who were at the end of gestation [81]. Furthermore, a study conducted in the USA highlighted the role of placental abruption in the association between elevated temperature and stillbirth; reporting a higher risk of stillbirths that occurred in warm months among pregnant women with placental abruption than those without placental abruption [37].

##### Maternal Stress

Lin et al. [53] reported that extreme temperature increases maternal stress during pregnancy, and this association may vary with the duration of sunlight exposure and pregnancy complications.

##### Cardiovascular Risk at Labour

A case crossover study conducted by Ha et al. [62] indicated that exposure to 1 °C increases in temperature during the last week of pregnancy increases the cardiovascular risk by 7% and that the risk was more evident on days closer to delivery.

##### Bacteriuria

A retrospective observational study conducted at a tertiary hospital in Doha, Qatar demonstrated increased maternal risk with significant bacteriuria, with high ambient monthly temperature (r = +0.677, *n* = 17, *p* = 0.003), and doubling rates noted in warm and drier months where the ambient temperature is 35 °C or greater (11.3% (at temperatures ≥ 35 °C) vs. 3.6% (at temperatures ≤ 35 °C; *p* < 0.0001 [87].

### 3.3. Elevated Temperature Exposure and Suceptible Gestational Periods

The authors synthesisedthe temperature variables and gestational exposure windows reported in 75 studies to identify whether any gestational period/s had been found to be particularly susceptible to high temperatures during pregnancy. Table A1 shows the various exposure windows examined in this scoping review.

Most of the studies examined multiple gestational windows. For example, some studies measured the effect of temperature over the entire gestation period (*n* = 19), while others examined short time frames. Most studies examined specific trimesters; first trimester (*n* = 22), second trimester (*n* = 23), and third trimester (*n* = 21). In addition, some studies focused on the last week of pregnancy (*n* = 21) or the last month of pregnancy (*n* = 16). Several studies also examined lag windows, and among them, 18 studies investigated the acute effect of heat exposure by examining the temperature on the day of delivery. In addition, 16 studies examined the effect of elevated ambient temperature during the first few weeks of pregnancy, while three investigated neonatal heat exposures from birth up to 28 days.

The exposure windows vary depending on the outcome being examined. Therefore, a critical window period for susceptibility to high temperature for adverse maternal, foetal, and neonatal outcomes could not be established. However, in general, findings indicate that the last few weeks of pregnancy may be associated with increased PTB and stillbirth risks. For instance, the most observed susceptible windows for PTB were acute exposures such as the last week (*n* = 11) [13,39,40,44,46,50,59,61,63,65,92], and the last month of pregnancy (*n* = 9) [13,57,59,61,65,68,79,90,92]. On the other hand, the most observed exposure window for stillbirths was the last week of pregnancy (*n* = 4) [15,36,49,91]. Outcomes such as PROM, placental abruption, neonatal deaths, cardiovascular events at labor, and maternal stress have a limited number of studies; therefore, susceptible window periods were not clear for those outcomes. In contrast, most of the LBW studies reported that LBW risk was elevated during the second and third trimester of pregnancy [35,55,78,94].

When examining congenital anomalies, five studies investigated maternal temperature exposure during the first eight weeks of pregnancy, as early pregnancy is a critical period of organogenesis [14,38,42,47,48]. For instance, heat exposure and neural tube defects were examined in the third and fourth gestational weeks [14]. Orofacial clefts and CHD were examined in the first eight weeks of gestation [38,42,47,48]. However, heat exposure and risk of hypospadias were investigated during gestational weeks 8–14 [85].

GDM studies examined multiple gestational-exposure windows, including the day of the OGTT, seven days before the OGTT, 14 days before the OGTT, 21 days before the OGTT, 28 days before the OGTT; 30 days before the OGTT; 35 days before the OGTT; and 56 days before the OGTT; with the OGTT at the 28th week of gestation [18,54,70]. For instance, a Spanish study reported a positive association between the mean ambient temperature on the day of the OGTT and 2−4 weeks before the routine gestational diabetes screening [54].A similar association was observed in the UK cohort, where a higher mean ambient temperature on the day of the OGTT showed an increased risk of being diagnosed with GDM [70]. In addition, Retnakaran et al. [18] revealed that maternal exposure to increasing ambient temperatures 3–4 weeks before the OGTT is associated with decreased beta-cell function and increased risk of GDM. Overall, reviewed literature shows great variation in susceptible windows among outcomes assessed.

## 4. Discussion

To our knowledge, this is the first scoping review addressing the relationship between maternal exposure to elevated ambient temperature and adverse maternal, foetal, and neonatal outcomes. Previous systematic reviews have summed up the evidence for associations between exposure to high temperatures and adverse birth outcomes, mainly focusing on three adverse birth outcomes: PTB, LBW, and stillbirth [9,20,21,27,29].We update those reviews, providing an overview of the available literature on this topic, covering eighteen adverse maternal, foetal, and neonatal outcomes.

### 4.1. Principal Findings

This scoping review employed a PRISMA extension for its scoping review method and identified 75 studies published between 2015 and 2020 that examined the impact of maternal exposure to elevated temperatures on maternal, foetal, and neonatal outcomes. Studies were conducted across 24 countries worldwide, but more than 70% were from North America and Asia. Temperature metrics and exposure windows varied considerably across studies and outcomes assessed. Diverse study designs were used, including retrospective, prospective, case-control, case-crossover, cross-sectional, and ecological, to assess the impact of elevated ambient temperature on:

(i) Eight types of adverse neonatal outcomes (PTB, LBW, stillbirth, neonatal mortality, neonatal morbidity, SGA, newborn telomere length, and newborn INR levels).
(ii) Three types of adverse foetal outcomes (congenital anomalies, reduced placental weight and volume and miscarriages), and
(iii) Seven types of adverse maternal outcomes (GDM, hypertensive disorders in pregnancy, placental abruption, PROM, cardiovascular events at labour, bacteriuria, and maternal stress).

PTB (*n* = 30) was the most common adverse outcome observed among reviewed studies. Other frequently reported heat-associated adverse outcomes are LBW and stillbirth.However, the relationship between elevated temperature and PTB appears stronger and more consistent than the other outcomes: more than 75% of the PTB studies (23 out of 30) reported a significant increase in PTB risk when pregnant women were exposed to elevated ambient temperatures. Moreover, over 40% of PTB studies reported that acute high temperature exposures (with a lag of 0–7 days) increased preterm delivery [12,39,40,44,50,52,59,61,63,65,76,77,92].

Similarly, studies that investigated the connection between high temperature and stillbirths also reported that the last week of pregnancy might increase the stillbirth risk [15,36,49,91]. However, most of the LBW studies (*n* = 9) examined longer exposure periods such as trimester-specific susceptible windows or the entire pregnancy period and found mixed results [10,35,41,55,71,78,88,92,94]. These findings are consistent with Chersich et al. [20], who conducted a systematic review and a meta-analysis to evaluate the impact of high temperatures on PTB, LBW, and stillbirth: reporting that heat associations with PTB and stillbirth appear strong, especially when pregnant mothers are exposed to elevated temperatures during their final weeks of pregnancy.

GDM is the most common adverse maternal outcome examined among reviewed studies. Studies consistently reported a positive association between elevated ambient temperatures and an increased risk for GDM, especially in summer [16,17,18,54,60,70,73,89]. This finding supports evidence from the previous systematic review conducted by Preston et al. [97], who reported a high prevalence of GDM and high pregnancy glucose levels in the summer months. In addition, our scoping review further investigated susceptible exposure windows for GDM as reported in the reviewed literature. We observed multiple susceptible windows, predominantly around the timing of OGTT. Also, we noted various diagnostic criteria for screening GDM, making it even harder to compare and conclude any particularly susceptible gestational period for heat-associated GDM.

Relatively few studies examined the heat-associated adverse foetal outcomes but among them, six reported the relationship between elevated temperature and congenital anomalies, namely: congenital heart defects, neural tube defects, hypospadias, and orofacial clefts [14,38,42,47,48,85]. Available evidence suggests that elevated temperature increases some types of congenital anomalies, such as congenital heart defects and neural tube defects [14,42,47,48]. Consistent with the recent systematic review findings on the effects of high temperatures on congenital anomalies [98], our scoping review also observed that the evidence was strongest for congenital heart defects. However, the susceptible exposure window period for congenital anomalies was less clear. Still, most studies (5 out of 6 studies) investigated the impact of heat on the first few weeks of pregnancy because the first trimester of pregnancy was considered the critical period of organogenesis [98]. However, due to the methodological diversity and the limited number of studies on each type of congenital anomaly, further research is required to conclude exposure relationships and susceptible windows for heat-associated congenital anomalies.

Mixed results were observed for heat-associated neonatal mortality, SGA, hypertensive disorders in pregnancy, miscarriages, placental abruption, and PROM. Further limited synthesis capability of evidence for some adverse outcomes, namely: reduced placental weight and volume, cardiovascular events at labour, bacteriuria, maternal stress, newborn telomere length, and newborn INR levels, means that it is too early to reach conclusions about these exposure relationships or susceptible window periods.

Lastly, all included studies reported potential confounding covariates, which may influence estimated associations between elevated ambient temperatures and maternal, foetal, and neonatal outcomes. The most commonly reported covariates include maternal demographic factors (maternal age, race/ethnicity, and marital status), maternal medical information (prepregnancy BMI, parity, conception season, pregnancy complications, and chronic comorbidity), lifestyle factors (maternal smoking and alcohol consumption), socioeconomic factors (maternal education, job, insurance status, and household income), infant sex, season, and air pollution [11,12,44,53,68,69,75,76].

### 4.2. Gaps and Recommendations for Future Research

One of the gaps identified from the scoping review findings is that, unlike PTB, LBW, and stillbirth, the evidence of heat-exposure associations on the other reported maternal, foetal, and neonatal outcomes were limited in global literature. These outcomes include congenital anomalies, neonatal mortality, neonatal morbidity, babies that are small for gestational age, reduced placental weight and volume, newborn telomere length, newborn INR levels, GDM, hypertensive disorders, miscarriages, placental abruption, premature rupture of membrane, cardiovascular events at labour, bacteriuria, and maternal stress. Among them, except for GDM, congenital anomalies, and neonatal mortality, all the other outcomes we reviewed had only one or two published articles examining the exposure relationship with high temperature. Due to this small number of studies, findings are inconsistent. Furthermore, the accuracy of the findings, i.e., estimates of effect size and exposure relationships for each outcome, may differ from the true accuracy, leading to bias. Therefore, we recommend more research on the less-reported outcomes identified from this scoping review to improve the consistency and accuracy of the exposure relationships and size of such effects towards better establishing the strength and coherence of evidence for this important area of environmental exposure.

Another gap identified from the reviewed studies is the lack of a clear definition of a heatwave; we found that heatwave definitions were inconsistent across studies. Studies used various heatwave definitions using the following characteristics: (i) temperature metrics (maximum temperature or mean temperature in most cases) [32,39,43,45] (ii) intensities (75th, 90th, 95th, or 98th percentile of mean temperature) [40,46], (iii) duration (2,3,4 days), and (iv) the number of heatwaves [32,40,41,42,43,46]. Studies adopted a combination of those four aspects to define heatwave or extreme temperature events. However, the key problem of using various definitions in exposure assessments is that the effect size may vary with the definition. Thus, it is not appropriate to compare the findings of different studies assessing the adverse maternal, foetal, and neonatal outcomes of heatwaves if they use different heatwave definitions. Therefore, future studies should consider conducting multicounty large studies and recommending a standardised use of the terms to enable more evidence synthesis of this important research area.

An identified gap of included studies is the small sample size for some outcomes. A small sample size was observed mainly in studies examining congenital anomalies [38,42,85], GDM [16,18,54,89], miscarriages [74,95], cardiovascular events [62], maternal stress [53], placental abruption [37], PROM [72], bacteriuria [87], reduced placental weight and volume [75], newborn INR values [51], and newborn telomere lengths [82]. Caution must be applied when interpreting findings from studies with a small sample size, as the findings might not be reliable or precise [99]. Therefore, future studies should be designed and conducted to employ sufficiently large sample sizes in order to have the power to obtain strong evidence of exposure relationships with those outcomes.

In addition, we observed a great inconsistency in study designs, temperature metrics, and exposure windows among the included studies, limiting the ability to compare the exposure effects between studies. This is consistent with discussion provided in previous reviews [20,21,27,29,100] conducted using epidemiological studies worldwide. However, to date, there is no general agreement regarding which temperature metrics would be applied for exposure assessments or which period of pregnancy would be more vulnerable to adverse outcomes. Therefore, future research should focus on sophisticated study designs and statistical methodologies for exposure assessments, as well as conducting more research on standardising temperature metrics and exploring susceptible exposure windows.

Furthermore, recent findings indicate that pregnant women start experiencing an increased risk of adverse birth outcomes when exposed to temperatures greater than 20 °C [101]. As temperature rises further, pregnant women are more susceptible to adverse birth outcomes, and the risk is more prominent after 30 °C [101]. Studies have reported that pregnant women are susceptible to sharp, sudden fluctuations in temperature; for instance, a few studies examined acute effects of temperature variability (i.e., temperature differences between the maximum and minimum temperatures) on adverse maternal, foetal, and neonatal outcomes [8,32,53,93,102,103,104].Among them, extreme temperature variability associated with elevated risk for PTB was the most common adverse birth outcome examined [32,93,102,104]. For instance, a recent study conducted in Shenzhen, China, reported that maternal exposure to extreme temperature variations over a short period increases PTB risk [102]. The study further revealed that the greatest temperature variability related to PTB risk arose in the second trimester of pregnancy, with 5.4% and 23.7% increased risk of PTB associated with 1 °C increase in intra-day temperature variability (i.e., temperature differences between the maximum and minimum daily temperatures), and inter-day temperature variability (i.e., the maximum increase in daily mean temperature between two neighboring days) respectively [102]. In addition, several studies reported that the association between acute exposure to extreme temperature variations was more robust to PTB; nevertheless, windows of susceptibility differ among studies [32,93,104]. However, the association between temperature variability and other maternal, foetal, and neonatal outcomes is still limited. For instance, Lin et al. [53] reported that increased temperature differences (daily maximum temperature–daily minimum temperature) were significantly associated with a higher level of emotional stress at a lag of 0–1 day and a lag of 0–2 days, and at lag day 0 and lag day 1. Yackerson et al. [103] reported that temperature variability is a risk factor for preterm premature rupture of membrane. However, Molina-Vega et al. [54] reported no impact of temperature variability on the glucose levels on the day of the OGTT or the preceding 14 and 28 days. These findings indicated that the current literature is insufficient to conclude the overalleffect of acute temperature variability and associated adverse maternal, foetal, and neonatal outcomes. Therefore, continued research focusing on temperature variability at multiple timescales may be crucial in understanding the impact of sudden temperature fluctuations on maternal, foetal, and neonatal health.

In addition, heat acclimatisation in pregnant women and the foetus may vary based on the diverse meteorological characteristics of the climate zones the pregnant women live in, but the evidence is limited. For instance, a study conducted in Israel reported that maternal exposure to extreme heat days during the cold season increases some types of congenital anomalies, such as atrial septal defects [8]. In contrast, a study conducted in Quebec, Canada reported that exposure to summer heatwaves (maximum daily temperatures ≥ 30 °C) was associated with an elevated risk of atrial septal defects [48]. The diversity in meteorological characters in climate zones may be the reason for this variation. For example, the Canadian study may have demonstrated an increased risk of congenital anomalies in warm months because pregnant women in temperate climate may not be acclimated to heat; thus, they are highly susceptible to heatwaves [48]. In contrast, Israel has a hot climate; therefore, pregnant women may be resilient to heat but less adapted to cold; thus, sudden temperature increases may affect pregnant women more during the cold season than the hot season [8]. Therefore, there is a possibility that pregnant women who consistently live in a hot climate may be more resilient to heat, while pregnant women in temperate climates are highly vulnerable to heat during the warm season. However, the underlying biological mechanism is not clear. Therefore, current evidence is insufficient to generate a conclusion; however, we recommend more research to address heat acclimatisation in pregnant women in different climate zones.

The lack of systematic reviews and meta-analyses for most of the outcomes assessed further restricts the ability to obtain more robust evidence on the impact of ambient temperature exposures on mothers, developing foetuses, and newborn babies. To date, only one meta-analysis was conducted on this topic, focusing on the high-temperature associations on PTB, LBW, and stillbirth, and found that evidence was most consistent and effect size largest for PTB and stillbirths [20]. In addition, two systematic reviews were conducted on GDM [97] and congenital anomalies [98] recently.Consistently with our scoping review, both of these systematic reviews highlighted that the low number of studies, statistical heterogeneity, and methodological diversity limits the possibility of conducting a meta-analysis to quantify the connection between temperature and the outcomes examined. Similarly, pooling the results of other outcomes mentioned in this scoping review is not feasible due to the considerable variation in study designs, sample size, temperature metrics, exposure windows, and heatwave definitions used to estimate the effect size: another gap identified in this review.

Furthermore, this scoping review found that the current knowledge of temperature-related adverse maternal, foetal, and neonatal effects is dominated by findings from the USA, China, Canada, and some European countries. Additionally, consistent with other systematic reviews [98,100], minimal studies were conducted in lower-middle-income countries and low-income countries. Therefore, we do not know the effect of heat on adverse pregnancy and birth outcomes in low-income countries. Furthermore, the findings are insufficient to provide an overall picture of the global research trends in this area and hinder the overall impact of heat on adverse maternal, foetal, and neonatal outcomes and associated regional differences. Moreover, heatwaves have become increasingly common due to climate change and are projected to increase in tropical regions, where most low- and middle-income countries are located. For instance, a review by Mora et al. [105] reported that countries in tropical areas such as parts ofSouth America, Africa, South Asia, Southeast Asia, and Northern Australia would be more exposed to deadly heatwave conditions in the coming decades. Similarly, a global review conducted by McElroy et al. [101] indicated increased risks of adverse birth outcomes across 14 low and middle-income countries in coming decades due to heat exposure. Corresponding to these findings, many of the countries in these areas have poor general health infrastructure and are also highly vulnerable to negative heat impacts, and research conducted in such settings is needed to provide useful insights to identify cost-effective strategies to mitigate the adverse pregnancy and birth effects of climate change.

Another gap identified is a lack of understanding of biological mechanisms potentially linking temperature with adverse outcomes. However, preliminary analysis of our scoping review shows that over 80% of reviewed studies proposed or added a discussion of a potential biological mechanism to explain their findings. Many studies argued that heat-stress-associated hormonal changes, heat-shock proteins, maternal dehydration, and altered thermoregulation mechanisms were responsible for adverse maternal, foetal, and neonatal outcomes [11,12,14,33,50,56,74]. However, none found an exact biological mechanism or experimented to understand the biological mechanisms of heat-associated adverse outcomes. Therefore, understanding the biological mechanism that links heat and adverse maternal, foetal, and neonatal outcomes remains an important research goal. The previous systematic review conducted by Sexton et al. [29] also emphasises the importance of understanding the biological mechanism and the contributing factors to fully recognise the effects or the dose–response relationship of maternal exposure to ambient temperature and stillbirths. We also recommend that future research is designed to explore the underlying biological mechanisms associated with adverse maternal, foetal, and neonatal outcomes mentioned in this scoping review. Furthermore, understanding these associations is critical to designing interventions to minimise heat-associated adverse outcomes among pregnant women and newborn babies.

### 4.3. Limitations of the Scoping Review

Several limitations must be highlighted. Firstly, we limited our search to the published articles from 2015–2020; we may have missed some studies before 2015 and after 2020. However, we noted that there is a dramatic increase in number of publications after 2015; thus, we provided the most up-to-date information on this topic. Secondly, we did not include hand-searched articles or grey literaturesuch as government reports, conference papers/proceedings, blogs, interviews, dissertations, and thesis; thus, we may have missed some studies that did not appear with our keyword search. Also, publication language was restricted to English only, as the authors could not access translation services. Thirdly, for this scoping review, we did not perform a quality appraisal for the included articles; thus, we are unable to comment on the quality of the studies and their findings; this may impact the interpretation of the findings of this review. Lastly, we did not examine the impact of cold temperatures on adverse maternal, foetal, and neonatal outcomes or effect modifiers such as air pollution, which were examined in some previous systematic reviews [21,106].

## 5. Conclusions

This review highlights the adverse impacts of heat on pregnant mothers, developing foetuses, and newborn babies, which is vital in developing appropriate public health interventions to reduce the burden of heat-related adverse maternal, foetal, and neonatal outcomes. It confirms the findings of existing reviews and adds to the existing knowledge by identifying gaps in outcomes, issues of inconsistent use of exposure metrics, gestational exposure windows and heatwave definitions. Current literature generally reported an increased risk of maternal exposure to heat for most outcomes examined. Our scoping review also identifies several gaps in the literature and provides recommendations for future research. In addition, our review has significant implications for public health and research in the future. The findings of this scoping review will update the overall knowledge of heat-associated adverse maternal, foetal, and neonatal impacts for pregnant women, health care professionals, and the community. Furthermore, for researchers, this scoping review guides the direction and design of future research studies and calls for more uniformity in exposure measures and designs, perhaps via purposefully designed multicountry large studies, to allow for improved analysis of this critical and emerging area of environmental exposure and birth and maternal outcomes.

## Figures and Tables

**Table 1:** Temperature metrics used among reviewed studies.

Temperature Exposure	Temperature Metrics	Number of Studies	Reference
Daily temperature exposure	Maximum daily temperature	26	[10,13,14,18,32,38,39,40,41,42,43,45,46,47,48,49,50,51,52,53,54,55,56,57,58,59]
	Minimum daily temperature	16	[10,13,18,39,41,43,50,51,52,53,54,55,56,57,58,59]
	Mean daily temperature	35	[15,16,18,32,41,46,51,52,53,54,55,56,57,59,60,61,62,63,64,65,66,67,68,69,70,71,72,73,74,75,76,77,78,79,80]
Weekly temperature exposure	Maximumweekly temperature	3	[14,48,81]
	Mean weekly temperature	4	[15,19,82,83]
Monthly temperature exposure	Maximum monthly temperature	2	[84,85]
	Mean monthly temperature	10	[17,51,52,84,85,86,87,88,89,90]
Hourly temperature exposure	Hourly ambient temperature	3	[91,92,93]
Trimester-specific exposures	Trimester-specific temperature	3	[75,76,94]
Apparent temperature exposure	Apparent temperature index	1	[32]
	Daily maximum apparent temperature (MAT)	2	[11,33]
	Mean apparent daily temperature	5	[12,34,35,36,37]
	Mean apparent weekly temperature	1	[35]
	Mean apparent monthly temperature	1	[35]
	Universal apparent maximum temperature (UATmax)	1	[38]
Heatindex/Heatwave/ Extreme heatevents exposure	Wet-bulb globe temperature (WBGT)	1	[95]
	Dry temperature	2	[44,58]
	Wet temperature	1	[58]
	Mean heat index (HI)	1	[59]
	Heatwave/extreme heat events	12	[6,32,38,39,40,41,42,43,44,45,46,47]

Note: Some studies examined exposure effects using multiple temperature metrics.

**Table 2:** Percentage of reviewed articles by season.

Season	Percentage
Studies conducted only in summer months or warm months, or hot months	34.67%
Studies conducted in both warm and cold months	25.33%
Studies conducted in the summer and spring months	2.67%
Studies conducted in all four seasons	22.67%
Season not stated	14.67%

## Appendix A

**Table A1:** Various exposure windows examined by reviewed articles.

Exposure Window	Number of Studies
Same day (Lag 0)	18
1 day before the case (Lag 0–1)	12
2 days before the case (Lag 0–2)	14
3 days before the case (Lag 0–3)	11
4 days before the case (Lag 0–4)	11
5 days before the case (Lag 0–5)	10
6days before the case (Lag 0–6)	10
Last week of pregnancy (Lag 0–7)	21
2 weeks before delivery (Lag 0–14)	4
3 weeks before delivery (0–21)	3
Last month of pregnancy (lag 0–28)	16
1st trimester	22
2nd trimester	23
3rd trimester	21
Entire pregnancy	19
Pre–conception(3 months prior to LMP)	8
1month prior to LMP	1
Conception date	1
0–8 weeks of pregnancy	1
Week 1 (0–7days) of gestation	2
Weeks 2–8	1
Weeks 3–4	1
Weeks 3–8	2
Weeks 8–14	1
1st month of gestation	6
2nd month of gestation	2
3rd month of gestation	2
Gestation week 20 to birth	1
0–36 weeks	2
Gestation week 22–26	1
Gestation week 27–29	1
Gestation week 30–32	1
Gestation week 33–35	1
Gestation week 36	1
Gestation week 37–38	1
Gestation week 39–43	1
0–28days after birth	3
Not stated	1

Note: Some studies examined more than one exposure window. LMP: last menstrual period.

## References

[1] Romanello M., McGushin A., Di Napoli C., Drummond P., Hughes N., Jamart L., Kennard H., Lampard P., Rodriguez B.S., Arnell N. (2021). The 2021 Report of the Lancet Countdown on Health and Climate Change: Code Red for a Healthy Future. Lancet.

[2] WHO (2021). COP26 Special Report on Climate Change and Health: The Health Argument for Climate Action.

[3] Perkins-Kirkpatrick S.E., Lewis S.C. (2020). Increasing Trends in Regional Heatwaves. Nat. Commun..

[4] Chambers J. (2020). Global and Cross-Country Analysis of Exposure of Vulnerable Populations to Heatwaves from 1980 to 2018. Clim. Chang..

[5] Åström D.O., Schifano P., Asta F., Lallo A., Michelozzi P., Rocklöv J., Forsberg B. (2015). The Effect of Heat Waves on Mortality in Susceptible Groups: A Cohort Study of a Mediterranean and a Northern European City. Environ. Health.

[6] Cil G., Cameron T.A. (2017). Potential Climate Change Health Risks from Increases in Heat Waves: Abnormal Birth Outcomes and Adverse Maternal Health Conditions. Risk Anal..

[7] Xu Z., Sheffield P.E., Su H., Wang X., Bi Y., Tong S. (2014). The Impact of Heat Waves on Children’s Health: A Systematic Review. Int. J. Biometeorol..

[8] Agay-Shay K., Friger M., Linn S., Peled A., Amitai Y., Peretz C. (2013). Ambient Temperature and Congenital Heart Defects. Hum. Reprod..

[9] Strand L.B., Barnett A.G., Tong S. (2011). The Influence of Season and Ambient Temperature on Birth Outcomes: A Review of the Epidemiological Literature. Environ. Res..

[10] Poeran J., Birnie E., Steegers E.A.P., Bonsel G.J. (2016). The Impact of Extremes in Outdoor Temperature and Sunshine Exposure on Birth Weight. J. Environ. Health.

[11] Asta F., Michelozzi P., Cesaroni G., De Sario M., Badaloni C., Davoli M., Schifano P. (2019). The Modifying Role of Socioeconomic Position and Greenness on the Short-Term Effect of Heat and Air Pollution on Preterm Births in Rome, 2001–2013. Int. J. Environ. Res. Public Health.

[12] Gronlund C.J., Yang A.J., Conlon K.C., Bergmans R.S., Le H.Q., Batterman S.A., Wahl R.L., Cameron L., O’Neill M.S. (2020). Time Series Analysis of Total and Direct Associations between High Temperatures and Preterm Births in Detroit, Michigan. BMJ Open.

[13] Li S., Wang J., Xu Z., Wang X., Xu G., Zhang J., Shen X., Tong S. (2018). Exploring Associations of Maternal Exposure to Ambient Temperature with Duration of Gestation and Birth Weight: A Prospective Study. BMC Pregnancy Childbirth.

[14] Auger N., Fraser W.D., Arbour L., Bilodeau-Bertrand M., Kosatsky T. (2017). Elevated Ambient Temperatures and Risk of Neural Tube Defects. Occup. Environ. Med..

[15] Ha S., Liu D., Zhu Y., Kim S.S., Sherman S., Grantz K.L., Mendola P. (2017). Ambient Temperature and Stillbirth: A Multi-Center Retrospective Cohort Study. Environ. Health Perspect..

[16] Chiefari E., Pastore I., Puccio L., Caroleo P., Oliverio R., Vero A., Foti D.P., Vero R., Brunetti A. (2017). Impact of Seasonality on Gestational Diabetes Mellitus. Endocr. Metab. Immune Disord. Drug Targets.

[17] Katsarou A., Claesson R., Ignell C., Shaat N., Berntorp K. (2016). Seasonal Pattern in the Diagnosis of Gestational Diabetes Mellitus in Southern Sweden. J. Diabetes Res..

[18] Retnakaran R., Ye C., Kramer C.K., Hanley A.J., Connelly P.W., Sermer M., Zinman B. (2018). Impact of Daily Incremental Change in Environmental Temperature on Beta Cell Function and the Risk of Gestational Diabetes in Pregnant Women. Diabetologia.

[19] Xiong T., Chen P., Mu Y., Li X., Di B., Li J., Qu Y., Tang J., Liang J., Mu D. (2020). Association between Ambient Temperature and Hypertensive Disorders in Pregnancy in China. Nat. Commun..

[20] Chersich M.F., Pham M.D., Areal A., Haghighi M.M., Manyuchi A., Swift C.P., Wernecke B., Robinson M., Hetem R., Boeckmann M. (2020). Associations between High Temperatures in Pregnancy and Risk of Preterm Birth, Low Birth Weight, and Stillbirths: Systematic Review and Meta-Analysis. BMJ.

[21] Zhang Y., Yu C., Wang L. (2017). Temperature Exposure during Pregnancy and Birth Outcomes: An Updated Systematic Review of Epidemiological Evidence. Environ. Pollut..

[22] Blencowe H., Cousens S., Chou D., Oestergaard M., Say L., Moller A.-B., Kinney M., Lawn J., The Born Too Soon Preterm Birth Action Group (2013). Born Too Soon: The Global Epidemiology of 15 Million Preterm Births. Reprod. Health.

[23] Walani S.R. (2020). Global Burden of Preterm birth. Int. J. Gynecol. Obstet..

[24] World Health Organization News: Preterm Birth. http://www.who.int/news-room/fact-sheets/detail/preterm-birth.

[25] WHO (2014). Global Nutrition Targets 2025: Low Birth Weight Policy Brief.

[26] Hug L., You D., Blencowe H., Mishra A., Wang Z., Fix M.J., Wakefield J., Moran A.C., Gaigbe-Togbe V., Suzuki E. (2021). Global, Regional, and National Estimates and Trends in Stillbirths from 2000 to 2019: A Systematic Assessment. Lancet.

[27] Bekkar B., Pacheco S., Basu R., DeNicola N. (2020). Association of Air Pollution and Heat Exposure with Preterm Birth, Low Birth Weight, and Stillbirth in the US. JAMA Netw. Open.

[28] Carolan-Olah M., Frankowska D. (2014). High Environmental Temperature and Preterm Birth: A Review of the Evidence. Midwifery.

[29] Sexton J., Andrews C., Carruthers S., Kumar S., Flenady V., Lieske S. (2021). Systematic Review of Ambient Temperature Exposure during Pregnancy and Stillbirth: Methods and Evidence. Environ. Res..

[30] Ouzzani M., Hammady H., Fedorowicz Z., Elmagarmid A. (2016). Rayyan—A Web and Mobile App for Systematic Reviews. Syst. Rev..

[31] World Bank Data Help Desk World Bank Country and Lending Groups. https://datahelpdesk.worldbank.org/knowledgebase/articles/906519-world-bank-country-and-lending-groups.

[32] Mohammadi D., Naghshineh E., Sarsangi A., Sakhvidi M.J.Z. (2019). Environmental Extreme Temperature and Daily Preterm Birth in Sabzevar, Iran: A Time-Series Analysis. Environ. Health Prev. Med..

[33] Schifano P., Asta F., Dadvand P., Davoli M., Basagana X., Michelozzi P. (2016). Heat and Air Pollution Exposure as Triggers of Delivery: A Survival Analysis of Population-Based Pregnancy Cohorts in Rome and Barcelona. Environ. Int..

[34] Basu R., Pearson D., Sie L., Broadwin R. (2015). A Case-Crossover Study of Temperature and Infant Mortality in California. Paediatr. Perinat. Epidemiol..

[35] Basu R., Rau R., Pearson D., Malig B. (2018). Temperature and Term Low Birth Weight in California. Am. J. Epidemiol..

[36] Basu R., Sarovar V., Malig B.J. (2016). Association between High Ambient Temperature and Risk of Stillbirth in California. Am. J. Epidemiol..

[37] Rammah A., Whitworth K.W., Han I., Chan W., Hess J.W., Symanski E. (2019). Temperature, Placental Abruption and Stillbirth. Environ. Int..

[38] Soim A., Sheridan S.C., Hwang S., Hsu W., Fisher S.C., Shaw G.M., Feldkamp M.L., Romitti P.A., Reefhuis J., Langlois P.H. (2018). A Population-Based Case-Control Study of the Association between Weather-Related Extreme Heat Events and Orofacial Clefts. Birth Defects Res..

[39] Arroyo V., Diaz J., Ortiz C., Carmona R., Sáez M., Linares C. (2016). Short Term Effect of Air Pollution, Noise and Heat Waves on Preterm Births in Madrid (Spain). Environ. Res..

[40] Ilango S.D., Weaver M., Sheridan P., Schwarz L., Clemesha R.E., Bruckner T., Basu R., Gershunov A., Benmarhnia T. (2020). Extreme Heat Episodes and Risk of Preterm Birth in California, 2005–2013. Environ. Int..

[41] Lawrence W.R., Soim A., Zhang W., Lin Z., Lu Y., Lipton E.A., Xiao J., Dong G.-H., Lin S. (2021). A Population-Based Case–Control Study of the Association between Weather-Related Extreme Heat Events and Low Birthweight. J. Dev. Orig. Health Dis..

[42] Lin S., Lin Z., Ou Y., Soim A., Shrestha S., Lu Y., Sheridan S., Luben T.J., Fitzgerald E., Bell E. (2018). Maternal Ambient Heat Exposure during Early Pregnancy in Summer and Spring and Congenital Heart Defects—A Large US Population-Based, Case-Control Study. Environ. Int..

[43] Mathew S., Mathur D., Chang A.B., McDonald E., Singh G.R., Nur D., Gerritsen R. (2017). Examining the Effects of Ambient Temperature on Pre-Term Birth in Central Australia. Int. J. Environ. Res. Public Health.

[44] Smith M.L., Hardeman R.R. (2020). Association of Summer Heat Waves and the Probability of Preterm Birth in Minnesota: An Exploration of the Intersection of Race and Education. Int. J. Environ. Res. Public Health.

[45] Wang J., Tong S., Williams G., Pan X. (2019). Exposure to Heat Wave during Pregnancy and Adverse Birth Outcomes: An Exploration of Susceptible Windows. Epidemiology.

[46] Wang Q., Li B., Benmarhnia T., Hajat S., Ren M., Liu T., Knibbs L.D., Zhang H., Bao J., Zhang Y. (2020). Independent and Combined Effects of Heatwaves and PM2.5 on Preterm Birth in Guangzhou, China: A Survival Analysis. Environ. Health Perspect..

[47] Zhang W., Spero T.L., Nolte C.G., Garcia V.C., Lin Z., Romitti P.A., Shaw G.M., Sheridan S.C., Feldkamp M.L., Woomert A. (2019). Projected Changes in Maternal Heat Exposure During Early Pregnancy and the Associated Congenital Heart Defect Burden in the United States. J. Am. Heart Assoc..

[48] Auger N., Fraser W.D., Sauve R., Bilodeau-Bertrand M., Kosatsky T. (2017). Risk of Congenital Heart Defects after Ambient Heat Exposure Early in Pregnancy. Environ. Health Perspect..

[49] Auger N., Fraser W.D., Smargiassi A., Bilodeau-Bertrand M., Kosatsky T. (2016). Elevated Outdoor Temperatures and Risk of Stillbirth. Int. J. Epidemiol..

[50] Cox B., Vicedo-Cabrera A.M., Gasparrini A., Roels H.A., Martens E., Vangronsveld J., Forsberg B., Nawrot T.S. (2016). Ambient Temperature as a Trigger of Preterm Delivery in a Temperate Climate. J. Epidemiol. Commun. Health.

[51] Iijima S., Sekii K., Baba T., Ueno D., Ohishi A. (2016). Seasonal Variation in the International Normalized Ratio of Neonates and Its Relationship with Ambient Temperature. BMC Pediatr..

[52] Li S., Chen G., Jaakkola J.J., Williams G., Guo Y. (2018). Temporal Change in the Impacts of Ambient Temperature on Preterm Birth and Stillbirth: Brisbane, 1994–2013. Sci. Total Environ..

[53] Lin Y., Hu W., Xu J., Luo Z., Ye X., Yan C., Liu Z., Tong S. (2017). Association between Temperature and Maternal Stress during Pregnancy. Environ. Res..

[54] Molina-Vega M., Gutiérrez-Repiso C., Muñoz-Garach A., Lima-Rubio F., Morcillo S., Tinahones F.J., Picón-César M.J. (2020). Relationship between Environmental Temperature and the Diagnosis and Treatment of Gestational Diabetes Mellitus: An Observational Retrospective Study. Sci. Total Environ..

[55] Ngo N.S., Horton R. (2016). Climate Change and Fetal Health: The Impacts of Exposure to Extreme Temperatures in New York City. Environ. Res..

[56] Ranjbaran M., Mohammadi R., Yaseri M., Kamari M., Yazdani K. (2020). Ambient Temperature and Air Pollution, and the Risk of Preterm Birth in Tehran, Iran: A Time Series Study. J. Matern. Neonatal Med..

[57] Vicedo-Cabrera A.M., Olsson D., Forsberg B. (2015). Exposure to Seasonal Temperatures during the Last Month of Gestation and the Risk of Preterm Birth in Stockholm. Int. J. Environ. Res. Public Health.

[58] Walfisch A., Kabakov E., Friger M., Sheiner E. (2017). Trends, Seasonality and Effect of Ambient Temperature on Preterm Delivery. J. Matern. Fetal Neonatal Med..

[59] Ward A., Clark J., McLeod J., Woodul R., Moser H., Konrad C. (2019). The Impact of Heat Exposure on Reduced Gestational Age in Pregnant Women in North Carolina, 2011–2015. Int. J. Biometeorol..

[60] Booth G.L., Luo J., Park A.L., Feig D.S., Moineddin R., Ray J.G. (2017). Influence of Environmental Temperature on Risk of Gestational Diabetes. Can. Med Assoc. J..

[61] Guo T., Wang Y., Zhang H., Zhang Y., Zhao J., Wang Y., Xie X., Wang L., Zhang Q., Liu D. (2018). The Association between Ambient Temperature and the Risk of Preterm Birth in China. Sci. Total Environ..

[62] Ha S., Nguyen K., Liu D., Männistö T., Nobles C., Sherman S., Mendola P. (2017). Ambient Temperature and Risk of Cardiovascular Events at Labor and Delivery: A Case-Crossover Study. Environ. Res..

[63] Ha S., Liu D., Zhu Y., Kim S.S., Sherman S., Mendola P. (2017). Ambient Temperature and Early Delivery of Singleton Pregnancies. Environ. Health Perspect..

[64] Ha S., Liu D., Zhu Y., Sherman S., Mendola P. (2018). Acute Associations between Outdoor Temperature and Premature Rupture of Membranes. Epidemiology.

[65] He J.-R., Liu Y., Xia X.-Y., Ma W.-J., Lin H.-L., Kan H.-D., Lu J.-H., Feng Q., Mo W.-J., Wang P. (2016). Ambient Temperature and the Risk of Preterm Birth in Guangzhou, China (2001–2011). Environ. Health Perspect..

[66] Jhun I., Mata D., Nordio F., Lee M., Schwartz J., Zanobetti A. (2017). Ambient Temperature and Sudden Infant Death Syndrome in the United States. Epidemiology.

[67] Junkka J., Karlsson L., Lundevaller E., Schumann B. (2021). Climate Vulnerability of Swedish Newborns: Gender Differences and Time Trends of Temperature-Related Neonatal Mortality, 1880–1950. Environ. Res..

[68] Liang Z., Lin Y., Ma Y., Zhang L., Zhang X., Li L., Zhang S., Cheng Y., Zhou X., Lin H. (2016). The Association between Ambient Temperature and Preterm Birth in Shenzhen, China: A Distributed Lag Non-Linear Time Series Analysis. Environ. Health.

[69] Liu X., Xiao J., Sun X., Chen Q., Yao Z., Feng B., Cao G., Guo L., He G., Hu J. (2020). Associations of Maternal Ambient Temperature Exposures during Pregnancy with the Risk of Preterm Birth and the Effect Modification of Birth Order during the New Baby Boom: A Birth Cohort Study in Guangzhou, China. Int. J. Hyg. Environ. Health.

[70] Meek C.L., Devoy B., Simmons D., Patient C., Aiken A.R., Murphy H.R., Aiken C.E. (2020). Seasonal Variations in Incidence and Maternal–Fetal Outcomes of Gestational Diabetes. Diabet. Med..

[71] Sun S., Spangler K.R., Weinberger K.R., Yanosky J.D., Braun J.M., Wellenius G.A. (2019). Ambient Temperature and Markers of Fetal Growth: A Retrospective Observational Study of 29 Million U.S. Singleton Births. Environ. Health Perspect..

[72] Song J., Lu J., Wang E., Lu M., An Z., Liu Y., Zeng X., Li W., Li H., Xu D. (2019). Short-Term Effects of Ambient Temperature on the Risk of Premature Rupture of Membranes in Xinxiang, China: A Time-Series Analysis. Sci. Total Environ..

[73] Su W.-L., Lu C.-L., Martini S., Hsu Y.-H., Li C.-Y. (2020). A Population-Based Study on the Prevalence of Gestational Diabetes Mellitus in Association with Temperature in Taiwan. Sci. Total Environ..

[74] Sun X., Luo X., Cao G., Zhao C., Xiao J., Liu X., Dong M., Wang J., Zeng W., Guo L. (2020). Associations of Ambient Temperature Exposure during Pregnancy with the Risk of Miscarriage and the Modification Effects of Greenness in Guangdong, China. Sci. Total Environ..

[75] Wang J., Liu X., Dong M., Sun X., Xiao J., Zeng W., Hu J., Li X., Guo L., Rong Z. (2020). Associations of Maternal Ambient Temperature Exposures during Pregnancy with the Placental Weight, Volume and PFR: A Birth Cohort Study in Guangzhou, China. Environ. Int..

[76] Wang Y.-Y., Li Q., Guo Y., Zhou H., Wang Q.-M., Shen H.-P., Zhang Y.-P., Yan D.-H., Li S., Chen G. (2020). Ambient Temperature and the Risk of Preterm Birth: A National Birth Cohort Study in the Mainland China. Environ. Int..

[77] Weng Y.-H., Yang C.-Y., Chiu Y.-W. (2018). Adverse Neonatal Outcomes in Relation to Ambient Temperatures at Birth: A Nationwide Survey in Taiwan. Arch. Environ. Occup. Health.

[78] Yitshak-Sade M., Novack L., Landau D., Kloog I., Sarov B., Hershkovitz R., Karakis I. (2016). Relationship of Ambient Air Pollutants and Hazardous Household Factors with Birth Weight among Bedouin-Arabs. Chemosphere.

[79] Zheng X., Zhang W., Lu C., Norbäck D., Deng Q. (2018). An Epidemiological Assessment of the Effect of Ambient Temperature on the Incidence of Preterm Births: Identifying Windows of Susceptibility during Pregnancy. J. Therm. Biol..

[80] Sun S., Weinberger K.R., Spangler K.R., Eliot M.N., Braun J.M., Wellenius G.A. (2019). Ambient Temperature and Preterm Birth: A Retrospective Study of 32 Million US Singleton Births. Environ. Int..

[81] He S., Kosatsky T., Smargiassi A., Bilodeau-Bertrand M., Auger N. (2018). Heat and Pregnancy-Related Emergencies: Risk of Placental Abruption during Hot Weather. Environ. Int..

[82] Martens D.S., Plusquin M., Cox B., Nawrot T.S. (2019). Early Biological Aging and Fetal Exposure to High and Low Ambient Temperature: A Birth Cohort Study. Environ. Health Perspect..

[83] Spolter F., Kloog I., Dorman M., Novack L., Erez O., Raz R. (2020). Prenatal Exposure to Ambient Air Temperature and Risk of Early Delivery. Environ. Int..

[84] Babalola O., Razzaque A., Bishai D. (2018). Temperature Extremes and Infant Mortality in Bangladesh: Hotter Months, Lower Mortality. PLoS ONE.

[85] Kilinc M.F., Cakmak S., Demir D.O., Doluoglu O.G., Yildiz Y., Horasanli K., Dalkilic A. (2016). Does Maternal Exposure during Pregnancy to Higher Ambient Temperature Increase the Risk of Hypospadias?. J. Pediatr. Urol..

[86] Karlsson L., Lundevaller E., Schumann B. (2019). The Association between Cold Extremes and Neonatal Mortality in Swedish Sápmi from 1800 to 1895. Glob. Health Action.

[87] Minisha F., Mohamed M., Abdulmunem D., El Awad S., Zidan M., Abreo M., Ahmad S., Fender G. (2019). Bacteriuria in Pregnancy Varies with the Ambiance: A Retrospective Observational Study at a Tertiary Hospital in Doha, Qatar. J. Périnat. Med..

[88] Molina O., Saldarriaga V. (2017). The Perils of Climate Change: In Utero Exposure to Temperature Variability and Birth Outcomes in the Andean Region. Econ. Hum. Biol..

[89] Vasileiou V., Kyratzoglou E., Paschou S.A., Kyprianou M., Anastasiou E. (2018). The Impact of Environmental Temperature on the Diagnosis of Gestational Diabetes Mellitus. Eur. J. Endocrinol..

[90] Yu X., Feric Z., Cordero J.F., Meeker J.D., Alshawabkeh A. (2018). Potential Influence of Temperature and Precipitation on Preterm Birth Rate in Puerto Rico. Sci. Rep..

[91] Kanner J., Williams A.D., Nobles C., Ha S., Ouidir M., Sherman S., Mendola P. (2020). Ambient Temperature and Stillbirth: Risks Associated with Chronic Extreme Temperature and Acute Temperature Change. Environ. Res..

[92] Son J.-Y., Lee J.-T., Lane K.J., Bell M.L. (2019). Impacts of High Temperature on Adverse Birth Outcomes in Seoul, Korea: Disparities by Individual- and Community-Level Characteristics. Environ. Res..

[93] Zhong Q., Lu C., Zhang W., Zheng X., Deng Q. (2018). Preterm Birth and Ambient Temperature: Strong Association during Night-Time and Warm Seasons. J. Therm. Biol..

[94] Ha S., Zhu Y., Liu D., Sherman S., Mendola P. (2017). Ambient Temperature and Air Quality in Relation to Small for Gestational Age and Term Low Birthweight. Environ. Res..

[95] Asamoah B., Kjellstrom T., Östergren P.-O. (2018). Is Ambient Heat Exposure Levels Associated with Miscarriage or Stillbirths in Hot Regions? A Cross-Sectional Study Using Survey Data from the Ghana Maternal Health Survey 2007. Int. J. Biometeorol..

[96] Wallace J., Horgan G., Bhattacharya S. (2012). Placental Weight and Efficiency in Relation to Maternal Body Mass Index and the Risk of Pregnancy Complications in Women Delivering Singleton Babies. Placenta.

[97] Preston E.V., Eberle C., Brown F.M., James-Todd T. (2020). Climate Factors and Gestational Diabetes Mellitus Risk–A Systematic Review. Environ. Health.

[98] Haghighi M.M., Wright C.Y., Ayer J., Urban M.F., Pham M.D., Boeckmann M., Areal A., Wernecke B., Swift C.P., Robinson M. (2021). Impacts of High Environmental Temperatures on Congenital Anomalies: A Systematic Review. Int. J. Environ. Res. Public Health.

[99] Hackshaw A. (2008). Small Studies: Strengths and Limitations. Eur. Respir. J..

[100] Kloog I. (2019). Air Pollution, Ambient Temperature, Green Space and Preterm Birth. Curr. Opin. Pediatr..

[101] McElroy S., Ilango S., Dimitrova A., Gershunov A., Benmarhnia T. (2021). Extreme Heat, Preterm Birth, and Stillbirth: A Global Analysis across 14 Lower-Middle Income Countries. Environ. Int..

[102] Li C., Bloom M.S., Lin S., Ren M., Hajat S., Wang Q., Zhang W., Ho H.C., Zhao Q., Lin Y. (2021). Temperature Variation and Preterm Birth among Live Singleton Deliveries in Shenzhen, China: A Time-to-Event Analysis. Environ. Res..

[103] Yackerson N., Piura B., Sheiner E. (2008). The Influence of Meteorological Factors on the Emergence of Preterm Delivery and Preterm Premature Rupture of Membrane. J. Perinatol..

[104] Wu M., Song L., Zheng X., Zhang L., Liu B., Wang L., Li H., Xiong C., Cao Z., Wang Y. (2019). Prenatal Exposure of Diurnal Temperature Range and Preterm Birth: FINDINGS from a Birth Cohort Study in China. Sci. Total Environ..

[105] Mora C., Dousset B., Caldwell I.R., Powell F.E., Geronimo R.C., Bielecki C.R., Counsell C.W.W., Dietrich B.S., Johnston E.T., Louis L.V. (2017). Global Risk of Deadly Heat. Nat. Clim. Chang..

[106] Poursafa P., Keikha M., Kelishadi R. (2015). Systematic Review on Adverse Birth Outcomes of Climate Change. J. Res. Med. Sci. Off. J. Isfahan Univ..

